# Enhancing the Potential of PHAs in Tissue Engineering Applications: A Review of Chemical Modification Methods

**DOI:** 10.3390/ma17235829

**Published:** 2024-11-27

**Authors:** Paweł Chaber, Silke Andrä-Żmuda, Natalia Śmigiel-Gac, Magdalena Zięba, Kamil Dawid, Magdalena Martinka Maksymiak, Grażyna Adamus

**Affiliations:** 1 Centre of Polymer and Carbon Materials, Polish Academy of Sciences, ul. M. Curie-Skłodowska 34, 41-819 Zabrze, Poland; sandra@cmpw-pan.pl (S.A.-Ż.); ngac@cmpw-pan.pl (N.Ś.-G.); kdawid@cmpw-pan.pl (K.D.); mmaksymiak@cmpw-pan.pl (M.M.M.); gadamus@cmpw-pan.pl (G.A.); 2 Department of Optoelectronics, Silesian University of Technology, ul. B. Krzywoustego 2, 44-100 Gliwice, Poland; magdalena.zieba@polsl.pl

**Keywords:** polyhydroxyalkanoates (PHAs), chemical modifications, surface modifications, polymer grafting, functionalized oligomers, tissue engineering

## Abstract

Polyhydroxyalkanoates (PHAs) are a family of polyesters produced by many microbial species. These naturally occurring polymers are widely used in tissue engineering because of their in vivo degradability and excellent biocompatibility. The best studied among them is poly(3-hydroxybutyrate) (PHB) and its copolymer with 3-hydroxyvaleric acid (PHBV). Despite their superior properties, PHB and PHBV suffer from high crystallinity, poor mechanical properties, a slow resorption rate, and inherent hydrophobicity. Not only are PHB and PHBV hydrophobic, but almost all members of the PHA family struggle because of this characteristic. One can overcome the limitations of microbial polyesters by modifying their bulk or surface chemical composition. Therefore, researchers have put much effort into developing methods for the chemical modification of PHAs. This paper explores a rarely addressed topic in review articles—chemical methods for modifying the structure of PHB and PHBV to enhance their suitability as biomaterials for tissue engineering applications. Different chemical strategies for improving the wettability and mechanical properties of PHA scaffolds are discussed in this review. The properties of PHAs that are important for their applications in tissue engineering are also discussed.

## 1. Introduction

Polyhydroxyalkanoates (PHAs) is a term given to a family of aliphatic polyesters synthesized by numerous bacteria and some archaea. Microorganisms accumulate PHAs in the form of intercellular granules that serve as carbon and energy storage material [1,2,3]. The accumulation of PHAs usually takes place when there is an excess of carbon sources with a concomitant deficiency of one (or more) of the following essential nutrients: nitrogen, oxygen, phosphorus, sulfur, or magnesium [4,5]. However, some microorganisms store PHAs even under non-limiting nutrient conditions [5]. So far, more than 300 strains of prokaryotes have been reported to produce biopolyesters [3,6]. Using the most diverse strains and growth media, it was possible to obtain bacterial polyesters, which together consist of more than 160 different monomeric units: starting from simple and saturated, through branched, unsaturated, and aromatic, and ending with those with additional functional groups, such as epoxy, halogen, or hydroxyl groups [7,8,9]. In general, bacteria store homo- or copolymers of hydroxycarboxylic acids, although terpolymers also occur [10].

**Figure 1:**
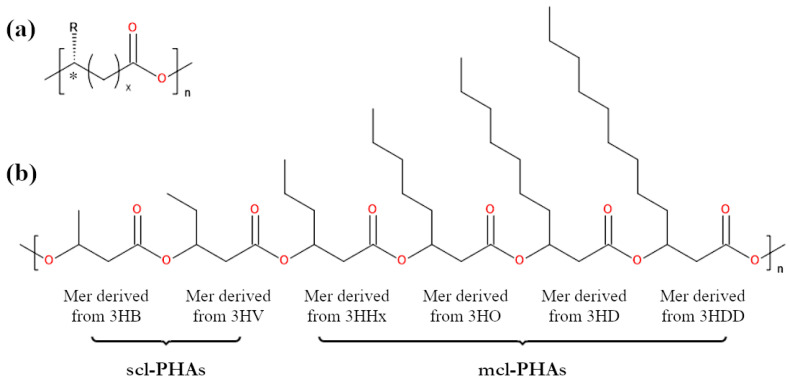
(**a**) General chemical structure of PHAs; * = chiral center, x = 1–4, R = hydrogen atom or an alkyl group. (**b**) The chemical structure of mers that make up scl-PHAs and mcl-PHAs; 3HB: (*R*)-3-hydroxybutyric acid (C_4_), 3HV: (*R*)-3-hydroxyvaleric acid (C_5_), 3HHx: (*R*)-3-hydroxyhexanoic acid (C_6_), 3HO: (*R*)-3-hydroxyoctanoic acid (C_8_), 3HD: (*R*)-3-hydroxydecanoic acid (C_10_), 3HDD: (*R*)-3-hydroxydodecanoic acid (C_12_).

From the chemical point of view, microbial polyesters are polymers in which the repeating units are derived from 3-, 4-, 5-, and 6-hydroxycarboxylic acids (Figure 1a). These units (the same or different) are linked to each other by ester bonds, which determine the chemical reactions that PHAs will undergo, including their biodegradation mechanism. It is also worth noting that it is mainly the presence of ester bonds in the main chain that makes biopolyesters biodegradable. Due to the differences in their chemical structure, biopolyesters can be divided into three groups: short-chain PHAs (scl-PHAs), medium-chain PHAs (mcl-PHAs), and long-chain PHAs (lcl-PHAs) [3,7,11]. In the first group, the chemical structure of the monomers consists of no more than five carbon atoms. In the second group, there are 6 to 14 carbon atoms, while the monomers of lcl-PHAs contain more than 14 carbon atoms [3,12]. Scl-PHAs include, among others, poly(3-hydroxybutyrate) (PHB) and its copolymer with 3-hydroxyvaleric acid (3HV), that is poly(3-hydroxybutyrate-*co*-3-hydroxyvalerate) (PHBV), which are two of the most important polymers among all PHAs. It is significant to note that PHB and PHBV are some of the best-studied, most widespread in nature, and the most commercially available representatives of bacterial polyesters. Scl-PHAs also include poly(3-hydroxybutyrate-*co*-4-hydroxybutyrate) (P34HB) and poly(4-hydroxybutyrate) (P4HB), while mcl-PHAs include those biopolyesters that, among others, consist of repeating units derived from 3-hydroxyhexanoic acid (3HHx) and 3-hydroxyoctanoic acid (3HO) (Figure 1b).

The most attractive feature of microbial polyesters is their outstanding biocompatibility. Numerous studies have shown that PHAs elicit a milder immune response than such widely used polyesters as polylactide (PLA), poly(lactide-*co*-glycolide) (PLGA), and polycaprolactone (PCL) [13,14,15,16]. The non-toxic nature of PHAs, combined with their ability to degrade under in vivo conditions, makes them highly promising materials for a range of biomedical applications.

These features have inspired researchers to explore the use of biopolyesters as matrices for controlled drug delivery and tissue engineering [17,18,19,20,21,22,23,24,25]. In this context, PHAs have proven useful in the engineering of, among others, soft tissues, blood vessels, heart valves, as well as cartilage, bone, and nerve tissues [20,21,26]. Moreover, biopolyesters are of particular interest in applications related to bone tissue regeneration due to their piezoelectric properties [27,28]. In addition to tissue scaffolds, PHAs could be used, for example, in the production of articular cartilage repair devices, cardiovascular patches, meniscus repair devices, orthopedic pins, screws, stents, sutures, and surgical meshes [20,25].

Although PHAs have many potential applications, products made from them are rarely found in everyday life. One of the few examples of the actual use of biopolyesters is a product called ‘TephaFLEX’, a resorbable surgical suture made of P4HB [20]. The reason for the lack of a wider industrial application of PHAs is still the high cost of their production, which is several times higher than that of petrochemical polymers [29]. Moreover, in medical applications, biopolyesters often do not exhibit adequate degradability and cytocompatibility. Therefore, PHAs undergo physical or chemical modifications to improve their biological properties.

**Figure 2:**
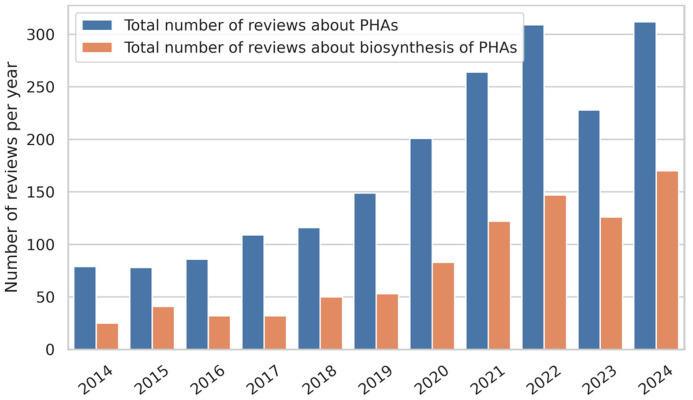
Bar chart illustrating the proportion of review articles on PHA production relative to the total number of review articles on these polyesters.

There are many review articles about PHAs, but they mainly deal with their biosynthesis in bacteria (Figure 2) [8,9,30,31,32]. Relatively few reviews have been devoted to the chemical modification of microbial polyesters, and even fewer to their modifications for tissue engineering purposes [33,34,35,36,37,38]. This paper aims to shed more light on this topic. In some regards, chemical methods are more reliable and beneficial than biotechnological and physical ones. Here, we summarize the literature describing the chemical methods used to improve the performance of PHAs as scaffold materials for tissue regeneration. We also provide basic information about the properties of PHAs that are essential for their application in regenerative medicine.

## 2. Properties of PHAs Important for Their Applications in Tissue Engineering

### 2.1. Mechanical Properties and Piezoelectricity

The mechanical properties of a scaffold are of great importance for its potential application in tissue engineering [39]. Such parameters of a scaffold as tensile strength and Young’s modulus dictate the types of tissue in which regeneration of that scaffold can participate. The ideal scaffold should have mechanical properties close to those of regenerated tissue. A rigid scaffold, for example, in contact with soft tissues, can cause damage to them and the subsequent onset of chronic inflammation in the host [40]. The reason for this is the lack of compatibility in terms of mechanical properties between the scaffold and the tissue. Therefore, flexible and elastic materials are preferentially used for soft tissue engineering, whereas stiff polymers are almost solely restricted to the regeneration of cartilage and bone tissues.

As mentioned earlier, PHAs are a group of polyesters exhibiting great structural diversity. Depending on bacterial culture conditions, PHA-producing strains can synthesize polyesters that consist of over 160 different monomeric units. This is a significant advantage of biopolyesters as it gives more options in selecting a polymer for a given application. However, only a few PHA polymers, such as PHB, PHBV, P34HB, P4HB, and PHBH, are commercially available and produced on a large scale [41]. This means that the range of mechanical properties offered by the most available PHA family members is often insufficient to meet demands in some tissue engineering approaches. Therefore, modifications of the chemical structure of PHAs are a way to expand their applications in regenerative medicine. The most important thermal and mechanical properties of selected representatives of PHAs are shown in Table 1.

When discussing the thermomechanical properties of microbial polyesters, it is typical to relate to their classification based on the chemical structure of the mers of which they are composed. Scl-PHAs are generally stiff and brittle and exhibit a relatively high degree of crystallinity (50–80%) [26,42,43]. On the other hand, mcl-PHAs are soft and flexible and less crystalline than scl-PHAs (X_c_ < 40%). Compared with scl-PHAs, mcl-PHAs are characterized by lower glass transition and melting temperatures, lower Young’s modulus values, and higher elongation at break [26]. In other words, mcl-PHAs are more elastic than scl-PHAs, and, hence, they are more frequently used in soft tissue engineering [44,45]. At the same time, stiff and highly crystalline PHB and PHBV are usually employed as scaffold materials for bone repair.

**Table 1:** Thermo-mechanical properties of selected representatives of PHAs [2,20,42,43,46,47,48,49].

Biopolyester	T_g_ [°C]	T_m_ [°C]	X_c_ [%]	E [GPa]	R_m_ [Mpa]	A [%]
PHB	5–10	173–180	60–80	3.5–4	20–40	3–8
PHBV 3%	8	170	59	2.9	38	5
PHBV 9%	N/A	162	N/A	1.9	37	N/A
PHBV 14%	N/A	150	N/A	1.5	35	N/A
PHBV 20%	−1	145	56	1.2	32	50
PHBV 25%	−6	137	54	0.7	30	100
PHBHHx 10%	−1	127	35	0.23	21	400
PHO	−38	49	25	0.017	N/A	300
P4HB	−50	53	<40	149	104	1000
P34HB 19–94%	−4 to (−46)	52–158	18–54	N/A	N/A	N/A
P34HB 3%	N/A	166	N/A	N/A	28	45
P34HB 10%	N/A	159	N/A	N/A	24	242
P34HB 16%	−7	130	43	N/A	26	444
P34HB 64%	−35	50	N/A	30	17	591
P34HB 90%	−42	50	N/A	100	65	1080

The percentage value given next to a specific PHA polymer indicates the molar content of the non-3HB monomer; T_g_, glass transition temperature; T_m_, crystal melting temperature; X_c_, degree of crystallinity; E, Young’s modulus; Rm, tensile strength; A, elongation at break; N/A, data not available.

PHAs exhibit a piezoelectric effect [50,51,52]. It is a desirable feature of biomaterials when they are utilized in regenerative medicine, especially in bone tissue engineering, since bone itself exhibits piezoelectric properties [53,54,55]. Piezoelectricity is a phenomenon that manifests itself by the generation of electrical charge on the surface of a material subjected to compressive or tensile forces [56]. It is a simple way to provide electrical stimuli to cells. Studies have shown that piezoelectric scaffolds, including those made of PHAs, promote bone tissue regeneration and formation [53,55,57,58].

### 2.2. In Vivo Degradability

Resorbability refers to the ability of a material to dissolve or disintegrate in physiological environments without causing any long-term complications [59]. Polymers resorb in the body either due to actions taken by the cells or through chemical dissolution. In vivo degradation of the material is an important property to consider during scaffold design. To achieve the therapeutic goal, the resorption rate of the scaffold should be similar to the regrowth rate of a tissue of interest [60]. Moreover, using a resorbable implant in tissue reconstruction allows the additional surgery required to remove the implant from the body once it fulfills its function to be avoided.

The degradation of biomaterials inside the body can occur enzymatically or non-enzymatically [61]. Hydrolysis is one of the most common non-enzymatic reactions that lead to bond breaking in a polymer. The susceptibility of a particular polymer to hydrolysis is most often studied through in vitro tests conducted under conditions that simulate physiological environments. Phosphate-buffered saline (PBS) or simulated body fluid (SBF) is usually used to mimic in vivo conditions. Studies using these buffers are very important—an absence of in vitro hydrolysis alongside in vivo degradation may suggest that the degradation within the body is due to increased cellular activity, including the presence of enzymes.

Doi et al. were among the first to study the hydrolytic degradation of PHAs using a phosphate buffer with a pH of 7.4 as the degradation medium [62]. They hydrolyzed polymer films of various PHAs, carrying out the degradation reaction at 37 °C for 200 days. The largest relative decrease in the number-average molecular weight (M_n_) was observed for a film produced from P34HB containing 16 mol% of 4-hydroxybutyrate units (referred to as P34HB 16%); a smaller decrease was recorded for P34HB 9%, while the smallest decrease was observed for PHB and PHBV 68%. Thus, these researchers showed that the degree of hydrolytic degradation of a biopolyester depends on its chemical composition. The differences in the rate of hydrolysis between the homopolymer of 3-hydroxybutyric acid and its copolymers are explained by the fact that the copolymers are characterized by much lower crystallinity than PHB. Studies have shown that hydrolysis in the amorphous regions of PHAs is up to 20 times faster than in the crystalline part [63].

In contrast to the average molecular weight, non-enzymatic hydrolysis of PHB under neutral conditions (pH~7) is usually not accompanied by mass loss (erosion) of the product made from this polymer [62,64,65,66]. A change in mass is not observed even when hydrolytic degradation is carried out for 730 days [67]. For this reason, bacterial polyesters are considered to be highly resistant to non-enzymatic hydrolysis.

It should be noted that products made from bacterial polyesters degrade to a greater extent when implanted into animals than when hydrolyzed in a PBS solution, which may indicate that enzymes are involved in the degradation of biopolyesters in vivo [64,68]. This can be investigated by performing the degradation of PHAs in vitro by adding an enzyme of animal origin to a phosphate buffer and comparing the results of hydrolysis conducted in the presence of this enzyme with those of non-enzymatic hydrolysis.

The degradation of PHAs caused by the presence of animal-origin enzymes can be studied in several ways. Although the results of such studies are not entirely conclusive, they tend to lean toward the fact that PHAs, with few exceptions, do not undergo hydrolysis catalyzed by animal enzymes [65,69,70,71]. It is important to highlight that, so far, no enzymes with PHA depolymerase activity—namely, enzymes exhibiting substrate specificity toward biopolyesters—have been found in human body fluids and tissues. Therefore, it has been assumed that non-specific lipases—a group of enzymes that break down fatty acid esters—may be responsible for the possible hydrolysis of PHAs in vivo [72]. These enzymes are commonly found in the human body, with pancreatic lipase being a typical representative [73]. For research, pancreatic lipase is most often obtained from the porcine pancreas and used in its pure form or as a mixture of enzymes called pancreatin, which contains lipase, amylase, and proteases [72].

Studies have shown that lipases produced by eukaryotic organisms significantly degrade only those PHAs which are derived from ω-hydroxyalkanoic acids, such as 3-hydroxypropionic acid (3HP), 4-hydroxybutyric acid (4HB), 5-hydroxyvaleric acid (5HV), and 6-hydroxyhexanoic acid (6HHx) [17,66,74,75]. Such biopolyesters do not contain side chains that act as substituents of the main chain. Among commercially available biopolyesters, only P34HB undergoes significant hydrolysis when catalyzed by animal-derived lipases. During the lipolytic hydrolysis of this copolymer, unlike the PHB homopolymer, a mass loss in the degradable film is observed [66,75,76].

As mentioned earlier, PHAs degrade faster in vivo than in vitro. The results of in vivo degradation tests depend on the monomeric composition of the biopolyester, the type of tissue in which the polymer is implanted, and the form of the implanted device (for example, whether the material is implanted as a film or fibers) [77]. In general, PHA copolymers, which have a lower degree of crystallinity than a PHB homopolymer, are more susceptible to in vivo degradation. This susceptibility is characterized by increased erosion and a greater decrease in the average molecular weight of the implanted copolymer [13,44,64,78]. Shishatskaya et al. studied the degradation of PHB and PHBV by implanting these polyester fibers into the muscular tissue of rats [64]. After 180 days, the average mass of the implanted fibers decreased to 74% of the initial mass for PHB and 65% for PHBV. Moreover, the authors indicated that enzymes in tissue fluid and immune cells, such as macrophages and foreign body giant cells (FBGCs), are responsible for the in vivo degradation of PHAs.

Other studies also pointed out that macrophages and FBGCs are primarily responsible for the in vivo degradation of PHAs [14,64,77,79,80]. The degradation of biopolyesters by macrophages and FBGCs is most often associated with the phagocytic function of these cells. Some researchers also suggest that reactive oxygen species (ROS) released by inflammatory cells, such as superoxide anion (O_2_^−^), hydrogen peroxide (H_2_O_2_), or nitric oxide (NO), may be responsible for the degradation of PHAs in vivo [69,81,82]. Lysosomal enzymes produced by macrophages and FBGCs are also indicated when considering the resorbability of PHAs [83,84].

In summary, bacterial polyesters are considered to be slow-degrading polymers under in vivo conditions. For this reason, it is suggested to use PHAs in applications where relatively high in vivo stability of the biodegradable polymer is required [69]. As mentioned earlier, the degradation time of the material used to design the scaffold should be consistent with the natural regeneration time of the target tissue. In most cases, however, the degradation time of commercially available biopolyesters is often too long, making their use in tissue engineering much more complicated [18,85].

### 2.3. Biocompatibility

Biocompatible materials do not induce an adverse immune-related reaction in contact with body fluids or tissues [86]. The lack of toxicity applies not only to the biocompatible polymer but also to its degradation products in vivo. Moreover, the body’s immune response cannot be strong enough to prevent the biomaterial from performing its therapeutic function. This property is especially important for PHAs, as these polyesters are characterized by very good biocompatibility compared to other polymers.

Bacterial cells are not the only places where biopolyesters naturally occur. Macromolecules containing no more than 200 3HB units have also been found in the cells of eukaryotic organisms [77,87]. This oligomeric PHB is referred to in the literature as “complexed PHB” (cPHB) [88]. It has been discovered in many tissues and organs of higher mammals (including humans), such as the blood, brain, heart, kidneys, nerves, eyes, and liver [77,87,89]. It has been shown that cPHB forms non-covalent complexes with inorganic polyphosphates, calcium ions, and proteins. These complexes function as ion channels embedded in the cell membrane, allowing inorganic ions to pass into the eukaryotic cell [77,89,90].

For a polymeric material to be described as biocompatible, the compounds formed as a result of its in vivo degradation (or the metabolites of these compounds) must also not be toxic to the host organism. The final product of PHB degradation, (*R*)-3-hydroxybutyric acid (3HB), similar to its oligomeric counterpart (cPHB), is found in the blood and tissues of both animals and plants [87,91,92]. It is also worth noting that in animals, this compound is a metabolite of fatty acid oxidation, which becomes the primary energy source in the case of blood glucose deficiency [91,93,94]. In other words, 3HB is classified as a so-called ketone body. Studies indicate that 3HB is not only a backup source of energy for animals, but also has many important functions as a regulatory molecule [90,91]. The most important of these functions is the inhibition of histone deacetylase and NLRP3 inflammasome activity [91,95,96]. It is also worth noting that the degradation products of other biopolyesters, such as PHBV, P34HB, and PHBHHx, are also non-toxic [42,97,98].

The fact that PHAs usually degrade slowly in the body has positive consequences. For example, PLA and PLGA degrade more rapidly, so that lactic acid (or glycolic acid), the end product of their in vivo degradation, begins to accumulate within the implant soon after implantation [99,100,101]. As a consequence, the tissue surrounding the implant becomes acidic, and a decrease in pH may accompany the implantation of PLA until it is completely absorbed [102,103,104,105]. This leads to a dangerous defensive reaction in the body, which is chronic inflammation (necrosis of host cells is also observed) [104,106,107]. Interestingly, the water-soluble oligomers formed during the degradation of PLA and PGA may also contribute to the aforementioned pathological process [108,109]. Inflammation caused by the presence of acidic degradation products is a significant medical problem, often limiting the use of PLA and its copolymers for therapeutic purposes [87]. In the case of bacterial polyesters, this problem is unlikely to occur. The use of PHB as a material for tissue scaffolds is also supported by the fact that the final product of its degradation, 3HB, is less acidic (pK_a_ = 4.70) than lactic acid (pK_a_ = 3.86) or glycolic acid (pK_a_ = 3.87) [110].

Tests to assess the biocompatibility of a given polymer in a specific application are carried out either under laboratory conditions (in vitro) or on animal models (in vivo) [86,111]. One of the most basic in vitro tests is the cytotoxicity test. This test determines whether the material causes cell death due to direct contact (direct contact assay) or as a result of the release of toxic substances (extract test) [111,112]. Both direct contact and extract tests have shown that biopolyesters are non-toxic to fibroblasts and osteoblasts [113,114,115]. Other in vitro studies have revealed that PHAs are not teratogenic or carcinogenic [42,116,117,118].

In vivo biocompatibility studies involve implanting material into a model animal organism and characterizing the immune system’s response to the implant, known as the tissue response [13,14,19,86,119,120,121,122]. In the immune response to the implant, there is a cascade of events that follow one another, although they can also co-occur. These stages include, among others, the onset of acute inflammation, the onset of chronic inflammation, the reaction to a foreign body, and the encapsulation of the implant with connective tissue (formation of a fibrous capsule around the foreign body) [86,123,124]. It should be noted that the duration and intensity of these processes determine the biocompatibility of the implant [86].

The intensity of the immune response to PHAs depends on the chemical purity of the implanted polyester [125,126]. Biopolyesters produced by bacteria may contain cell residues, such as bacterial lipopolysaccharides and proteins, which are endotoxins that can cause fever, a systemic symptom of inflammation [18,125,126]. Studies indicate that PHAs of high purity induce a milder immune response than PLA, PLGA, or PCL [13,14,15,16]. This less intense tissue response is characterized by (i) reduced immune cell infiltration, (ii) the induction of cytokine expression that promotes angiogenesis and implant acceptance, for example, by macrophage polarization towards the M2 phenotype, and (iii) the absence of a fibrous capsule or a smaller capsule thickness [40,77,123]. It should also be emphasized that PHAs have better biocompatibility, even though their in vivo degradation proceeds more slowly than that of PLA and PLGA. This remains true despite the fact that the capsule formed around the PHA implant may persist until the material is completely degraded [77].

Studies using PHB homopolymer and its copolymers indicate that these generally do not differ in tissue response [78,84]. Some reports, however, suggest that PHA copolymers are better tolerated by the host than the PHB homopolymer [14,84,121]. What distinguishes PHB from its copolymers is the in vivo degradation rate and mechanical properties. Neither of these differences is beneficial for PHB, as it degrades more slowly and is less flexible than its copolymers [14]. Sometimes the slowly degrading polymer, remaining at the implantation site for a relatively long time, leads to chronic inflammation [127]. The adverse effect of the prolonged presence of PHB implants is most often explained by the fact that they are too rigid, thus subjecting the adjacent tissues to constant mechanical stimulation [14,84]. This stimulation arises from a lack of compatibility in mechanical properties between the implant and the tissue, and often results in damage to the latter, which also has an impact on the immune system [40].

Studies have shown that bacterial polyesters, in addition to very good biocompatibility, also exhibit high hemocompatibility [126]. Therefore, they could potentially be used for the production of medical devices that come into contact with blood.

### 2.4. Cytocompatibility

Cytocompatibility is a concept related to the assessment of the biocompatibility of a material. However, it has a broader meaning than cytotoxicity. Cytocompatible materials do not cause cell death or negatively affect the structure and function of the tissues with which they come into direct contact [128]. A polymer can therefore be non-cytotoxic and, at the same time, non-cytocompatible, but not the other way around; it may not cause cell death while preventing the cells from performing certain functions. The basic tests assessing cytocompatibility in relation to a specific type of cells are the test of adhesion of these cells to the surface of the tested material and the test of their multiplication (proliferation) [128]. When examining cytocompatibility, attention is also paid to the morphology of cells adhering to the material’s surface. The closer their shape is to the natural one, the more cytocompatible the material is [128].

Cytocompatibility is a property of polymers that is extremely important in the context of their use in the production of tissue scaffolds. It defines the types of tissue in which the polymer can participate in the regeneration process. Studies described in the literature have shown that PHAs promote the adhesion and proliferation of many types of animal cell. These include fibroblasts, osteoblasts, chondrocytes, cardiomyocytes, Schwann cells, mesenchymal stem cells, smooth muscle cells, keratinocytes, and neural stem cells [15,113,114,129,130,131,132,133,134,135].

## 3. Applications of PHAs in Tissue Engineering

As mentioned earlier in this review, PHAs are characterized as biocompatible and biodegradable polyesters. This makes them promising candidates for tissue engineering applications [136]. It is worth noting here that one of the main goals of biomaterials in regenerative medicine should be to replace or repair damaged tissues. Therefore, they should, above all, enhance the healing of damaged organs through suitable mechanical properties and have an appropriate surface topography to support cell growth and adhesion [137,138].

**Figure 3:**
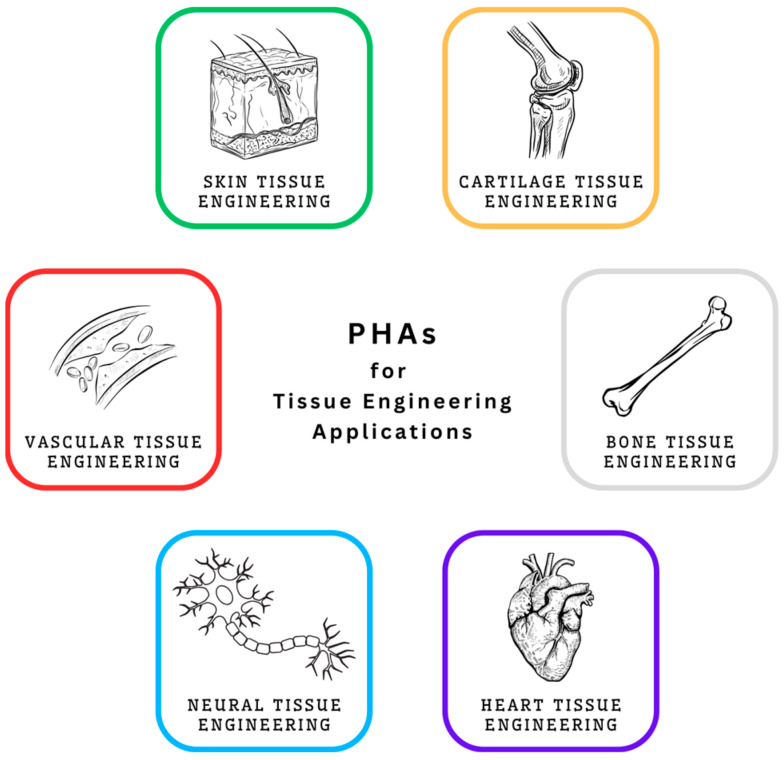
Applications of microbial polyesters in various areas of tissue engineering. The authors were inspired by Ref. [21] in the preparation of this figure.

There are many attempts described in the literature to apply PHAs in the field of tissue engineering (Figure 3), primarily involving the use of PHB, PHBV, P34HB, P4HB, PHBHHx, and PHO [139,140]. Interestingly, PHAs are being studied for both soft tissue and hard tissue engineering applications [44]. For this purpose, one possibility is to prepare three-dimensional (3D) scaffolds that provide support for intercellular connections, which may result in the formation of an extracellular matrix and constitute a carrier for growth factors and cytokines at the repair site of the damaged tissue [141]. Additionally, studies were also carried out on nanofiber membranes, films, patches, conduits, and strips [44].

In the case of the hard tissue engineering field, PHB could meet the requirements of biomaterials for bone tissue regeneration exceptionally well. This is, among other reasons, due to such properties as high crystallinity, bioresorbability, and relatively long in vivo degradation time. Moreover, PHB is characterized by piezoelectric properties similar to those found in natural bones [142]. However, there are some limitations in the use of this biopolyester for bone regenerative applications. This is the reason why many attempts are made to blend PHB with other biopolymers, for example, with PHBV or PCL [113,143]. The regeneration of cartilage tissue with the use of PHAs was also studied by researchers. Articular cartilage is primarily responsible for movements without fractions, but arthritis or various injuries can damage this tissue. Interestingly, researchers observed that PHBHHx/PHB scaffolds support the growth of chondrocytes much better than those made only of PHB [144]. Moreover, Petrovova et al. showed that PHB/chitosan porous biomaterial promoted the formation of hyaline-like cartilage, which supported the osteochondral regeneration [145].

Studies have shown that PHAs have also great potential in the soft tissue engineering field. It should be noted that these studies typically include mcl-PHAs, due their elastomeric properties and low-melting point [45]. In the literature, we can find examples of the use of PHAs in the form of stents, wound dressings, cardiac patches, blood vessels, heart valves, and nerve conduits [44,45]. Tissue-engineered blood vessels and cardiac valves constitute an interesting approach to their synthetic polymeric counterparts. In the case of the latter, often thrombosis and infections occur, and these limit their application and promote the use of biocompatible vessel grafts with growth potential. For example, PGA coated with P4HB has suitable properties for heart valve and vascular tissue engineering approaches, while PHO could be used for the production of heart patches [146]. In neural tissue engineering, piezoelectric biomaterials show great potential due to their ability to generate piezoelectric surface charges. However, PHB is characterized as too stiff to mimic native nerves. Therefore, PHB is blended with other polymers (for example, PCL, PLLA, chitosan). Sometimes other copolymers such as PHBHHx, PHBV, or P34HB are used instead [147]. Biomaterials for skin tissue engineering applications should, especially, be flexible and elastic, but also strong to support the proliferation of keratinocytes, fibroblasts, and melanocytes [148]. One of the interesting attempts to use PHAs in this field was described by Sankar et al. They fabricated a 3D hydrogel chitin/PHBV microporous scaffold, whose properties supported the adhesion and proliferation of human dermal fibroblasts (HDF). Moreover, this scaffold had a controlled swelling ratio and its biodegradation was suitable for wound dressings [149].

Overall, it can be concluded that PHAs and their blends show great potential in the field of hard and soft tissue engineering. However, further studies are needed to improve the properties of these biomaterials, making them more suitable for specific tissue engineering applications.

## 4. Chemical Modifications of PHAs

Biopolyesters possess many desirable properties for tissue engineering applications. However, they are not free of drawbacks that limit their utility in regenerative medicine. The scaffolds made from PHB and PHBV, i.e., from the most important members of the PHA family, are brittle and stiff. The implantation of these scaffolds in an animal model can result in tissue damage and chronic inflammation due to the mismatch of mechanical properties between the tissue and the implant. The brittleness and stiffness of PHB and PHBV are due to their high degree of crystallinity (50–80%). The high content of the crystalline phase in PHB and PHBV also results in lowering their resorption rate. Fortunately, the crystallinity of PHAs can be reduced by modifying their chemical structure.

**Figure 4:**
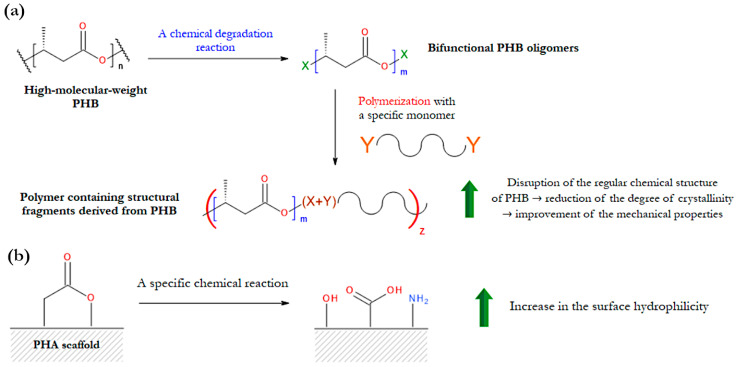
Two approaches are used for altering the chemical structure of microbial polyesters. (**a**) One utilizes polymerization of the PHB oligomers to obtain its multiblock copolymers, and (**b**) the second focuses on modifying the surface chemistry of the scaffold.

The problem with the crystallization behavior is particularly evident in the case of PHB scaffolds. The high tendency of PHB to crystallize is due to its orderly and regular chemical structure. This polyester, synthesized by many bacterial species, is an isotactic homopolymer; that is, it is made of repeating units of one type, all of which have the same (R) configuration on the tertiary carbon atom [150]. Thus, by disrupting the repetitive structure of PHB, it is possible to reduce its degree of crystallinity and improve its mechanical properties and degradability in vivo. This can be done through various chemical methods, which aim to modify the chemical structure of PHB. An illustrative example of this method is the copolymerization reaction involving PHB oligomers with non-PHB-derived monomers, such as 1,6-hexamethylene diisocyanate and poly(ethylene glycol) (PEG) (Figure 4a) [151,152]. The overall effect of this process is the introduction of other monomers into the backbone of the discussed homopolymer. This leads to the formation of multiblock copolymers containing structural segments derived from PHB. These copolymers have significantly lower crystallization ability than the PHB homopolymer [151,152].

Functional oligomers for polycondensation reactions can be obtained, among other ways, by conducting the degradation of PHA chains [151,152,153]. Interestingly, the degradation reaction itself is an example of a chemical modification method, as the chemical structure of the degraded polymer changes as a consequence of this reaction. In addition to their use in the synthesis of multiblock polymers, PHA oligomers can also be utilized to obtain di- and triblock copolymers [154,155].

Another drawback of microbial polyesters, which hinders their widespread use in tissue engineering, is the lack of structural features that promote an appropriate cellular response upon contact with them [36,156,157,158]. The term “cellular response” refers to biological processes such as cell adhesion, proliferation, migration, and differentiation. In other words, PHAs are hydrophobic [159]. Given that the surface properties of scaffolds significantly impact cell behavior, a simple and effective way to improve the cytocompatibility of PHAs is to modify only the surface of the medical devices made from them (Figure 4b) [35,160]. Many surface modification methods have been developed to improve the hydrophilicity of PHA scaffolds [35].

Surface modification techniques are generally divided into chemical and physical methods [160,161,162]. The second ones are based on various physical phenomena, such as adsorption from a solution onto a solid surface and gas ionization by electrical discharge, occurring during a transition into the plasma state. The physical methods include, among others, dip coating, the formation of self-assembled monolayers (SAMs), and the production of plasma by dielectric barrier discharge and corona discharge [160,163]. These methods are commonly used to improve the biological properties of PHAs [35,164,165,166]. However, they have at least one serious drawback: the low structural stability of the physically modified surfaces [162,167]. Chemically modified surfaces, on the contrary, are characterized by high long-term stability [168]. Moreover, the functional groups formed due to chemical reactions can serve as substrates for further chemical modifications, including protein immobilization [160,169]. Studies have shown that the binding of specific proteins or signaling molecules to the surface of a scaffold can considerably improve its therapeutic efficacy [35,160,170]. The graft copolymerization reaction is one of the most commonly used reactions for chemical surface modification.

### 4.1. Graft Copolymers of PHAs

Graft copolymerization is a reaction in which the main chain of one polymer is attached to the chain of another polymer. Subjecting microbial polyesters to grafting results in branched or crosslinked copolymers that differ significantly in physicochemical properties from their linear precursors. Grafting reactions are widely used to modify the hydrophilicity of PHAs by introducing polar groups into the main chain, such as –COOH, –OH, and –NH_2_. They can also be used to covalently bond synthetic polymers, leading to entirely new types of materials with various properties [171].

The combination of two polymers and the formation of a graft copolymer occur through so-called active centers [172]. These centers are located along the chains, where branches are attached. Active centers can be of two types: ionic or radical [172,173,174]. For this reason, graft copolymerization is classified into radical, controlled (living) radical polymerization, anionic, and cationic types [173,175]. In this study, only the grafting of polymers proceeding through radical mechanisms will be discussed, as the vast majority of graft copolymerization reactions described in the literature, involving the grafting of PHA chains, occur through radicals.

To generate radical active centers, radiation of a specific wavelength or chemical reagents, known as radical reaction initiators, are most commonly used [172,173]. Ultraviolet (UV) light, electron beams, or gamma rays can be utilized as a radiation source [172]. Without going into details, it can be stated that the interaction of radiation with polymers leads to the homolytic cleavage of their bonds and the formation of macroradicals [175,176]. A chemical property of PHAs that allows them to participate in radical reactions is the relatively easy removal of a hydrogen atom from their structure. The cleavage of the C–H bond is associated with the formation of radicals [177].

In the chemical structure of the repeating unit of PHB, three types of hydrogen atoms can be distinguished: primary (hydrogen atoms of the methyl group, –CH_3_), secondary (hydrogen atoms of the methylene group, –CH_2_–), and tertiary (the hydrogen atom of the methine group, >CH–). It has been shown that the removal of a hydrogen atom is inversely proportional to the number of hydrogen atoms bonded to a carbon atom, meaning that tertiary hydrogen atoms are the most reactive [178]. This can be reflected in the stability of each radical, with tertiary radicals being the most stable, followed by secondary, and primary radicals being the least stable [179]. This theory has been proven using electron paramagnetic resonance (EPR) spectroscopy [180]. It is worth mentioning that a small amount of secondary carbon radicals is produced during the reaction [35,181].

**Figure 5:**
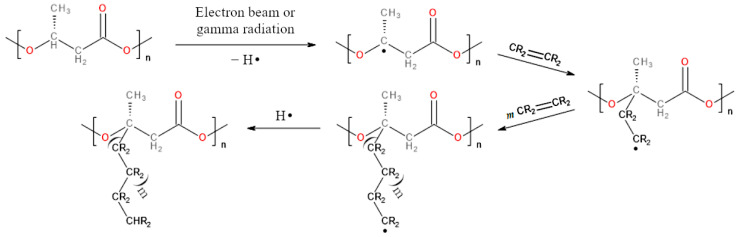
Scheme illustrating the stages of initiation, propagation, and termination of radical reactions involving PHB, which are induced by ionizing radiation. For simplicity, only one of the possible termination reactions is presented in the diagram.

Tertiary PHB radicals, formed during the initiation reaction, subsequently react with monomer molecules that typically contain a double bond. This leads to the formation of a C–C bond between PHB and the monomer, as well as the generation of a new radical that can react with another alkene molecule (undergoing propagation) [176,181,182,183,184]. As the propagation step is repeated multiple times, a graft copolymer is formed, with a chain of another polymer attached to the main chain of PHB. The stages of initiation, propagation, and termination of radical reactions induced by ionizing radiation, in which PHB serves as the polymer onto which various types of alkenes are grafted, are presented in Figure 5.

We can highlight two methods of grafting induced by ionizing radiation: the pre-irradiation method and the simultaneous method [35,172,175]. In the first method, only the polymer is irradiated to create active centers, after which the monomer is added (a two-step synthesis). In the second method, both the polymer and the monomer are in the solution or suspension while being irradiated (a one-pot synthesis). Both methods possess their advantages and disadvantages, as described by Pino-Ramos et al. [172].

To initiate the radical reaction, instead of using ionizing radiation, a chemical compound containing a chemical bond that relatively easily undergoes homolytic cleavage can be used [184]. The most typical examples of such compounds are peroxides, which contain labile –O–O– bonds. These bonds break at elevated temperatures, resulting in two radicals that initiate the graft copolymerization of PHB, primarily by abstracting a tertiary hydrogen atom from its macromolecules [180,183,185].

Methods that use UV radiation to initiate graft copolymerization are, in a sense, a combination of the previously described methods for initiating radical reactions, namely radiation methods and those using chemical polymerization initiators. Unlike electron beams and gamma radiation, UV light is not ionizing radiation [176]. This means it does not carry enough energy to break any of the C–H bonds in PHB and initiate graft copolymerization. For this reason, UV radiation is used in conjunction with so-called photoinitiators [176].

The most commonly used free radical initiator is benzophenone. After absorbing UV light, benzophenone gets excited into a singlet state, which then transforms into a triplet state due to intersystem crossing [186,187]. The excited molecule is capable of abstracting a hydrogen atom from the PHB chain, creating an active center, which may further undergo propagation.

In the synthesis of graft copolymers, in addition to typical laboratory glassware, extruders are also used, with their hoppers being filled with polymers, monomers, and radical initiators [180,188,189,190]. The chemical process taking place in the extruder is referred to as reactive extrusion. The biggest advantage of this method is that it does not require the use of solvents. Another advantage is good temperature control and excellent mixing of the reaction mixture compared to bulk grafting [190]. A graft copolymer of PHB and cellulose was synthesized using the reactive extrusion method [180]. The modification in the molten phase was carried out in the presence of dicumyl peroxide. PHB grafted onto a cellulose chain exhibited better thermal stability and lower crystallinity compared to its substrates [102]. XRD measurements also showed that the crystalline regions decreased in size. Large crystalline regions of PHB are the main cause of its brittleness [191,192].

A major advantage of methods based on graft copolymerization is that they can be relatively easily applied to modify the chemical structure of the surfaces of all types of polymer materials. Radical polymerizations can occur under such mild conditions that it is possible to modify the surface of electrospun fibers [35,157,193,194]. The aminolysis and acid hydrolysis reactions of biopolyesters, as discussed in the following paragraphs, are carried out at relatively high temperatures and over extended periods of time, which may negatively impact the fibers’ morphological and mechanical properties [33,169,195]. It is also possible to destroy the fibrous structure under these conditions [196]. Graft copolymerizations are a few examples of chemical reactions through which polar functional groups can be introduced onto the surface of PHA fibers.

Chen and co-workers employed radical reactions for surface modification in a slightly different way. In their method, the surface of a fibrous PHB membrane is activated using a radical initiator (hydrogen peroxide), however, the generated radicals undergo hydrolysis instead of copolymerization [170]. This method allows for the introduction of more hydroxyl moieties on the surface of the membrane. These groups were then used to initiate a series of polar reactions, resulting in the binding of platelet-rich plasma proteins to the surface of the PHB fibers. It was found that such modified membranes better support the proliferation of rabbit tenocytes compared to unmodified membranes [170].

**Figure 6:**
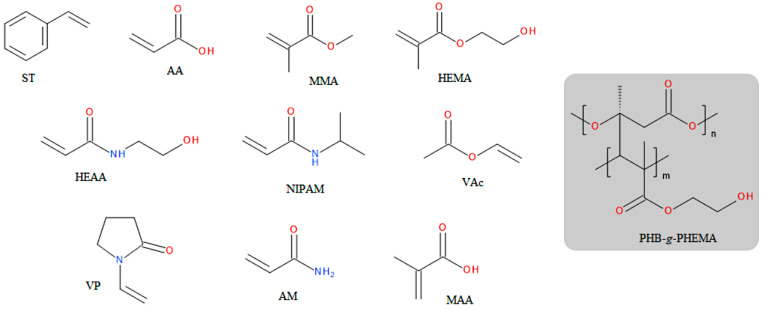
Chemical structures of the monomers used for grafting the surfaces of PHB and PHBV products, and the structure of the PHB-g-PHEMA copolymer.

Surfaces of PHAs modified with grafting reactions have gained increasing attention in recent years [35]. Using gamma radiation to generate PHA macroradicals, surface modification was applied to PHB and PHBV in the form of powders, PHB in the form of films, and electrospun PHBV fibers [181,193,197,198,199,200,201,202]. Modification of the powders was achieved using both the pre-irradiation method and the simultaneous radiation method. It was shown that the pre-irradiation method achieves better yields for homopolymers, whereas the simultaneous radiation method is more effective for copolymers [197,198,199]. Styrene (ST), acrylic acid (AA), methyl methacrylate (MMA), 2-hydroxyethyl methacrylate (HEMA), *N*-(2-hydroxyethyl)acrylamide (HEAA), *N*-isopropylacrylamide (NIPAM), and vinyl acetate (VAc) were used for the radiation-induced grafting of PHB and PHBV surfaces. The structures of the monomers and poly(3-hydroxybutyrate)-*g*-poly(2-hydroxyethyl methacrylate) (PHB-*g*-PHEMA) are shown in Figure 6.

Grafting PHAs with monomers such as AA, HEMA, and HEAA allows for the introduction of polar functional groups, such as hydroxyl and carboxyl groups, onto their surfaces. This results in a significant increase in the hydrophilicity of the modified surfaces, which, in turn, is expected to lead to better adhesion and proliferation of cells cultured on them [122]. Among the products mentioned in the previous paragraph, however, only PHBV fibers grafted with NIPAM were used for cell adhesion and viability studies [193]. It was found that adipose-derived stem cells cultured in the presence of NIPAM-modified fibers adhered more strongly to their surfaces than to unmodified fibers [193]. Nevertheless, viability studies showed that NIPAM-grafted fibers supported the proliferation of these cells only slightly better than non-grafted fibers.

The copolymer PHB-*g*-PHEAA underwent indirect biological studies, as it was chemically modified before these experiments. The modification led to the production of polyurethane foam [181]. However, these studies were somewhat limited; the viability of human fibroblast cell lines was assessed solely for the polyurethane scaffold, without evaluating the viability of the unmodified PHB. Consequently, the effects of these modifications on cell–surface interactions were not examined. Despite this, the authors concluded that PHB-based polyurethane foams are suitable for scaffolds in skin defect regeneration [181].

A major disadvantage of surfaces modified by grafting, particularly relevant to the needs of tissue engineering, is the reduced degradability of the materials [197,198,201]. Wada et al. showed that PHB grafted with AA or VAc completely lost its ability to undergo enzymatic degradation when the degree of grafting exceeded 5% [201,202]. However, it was possible to restore the biodegradability of the grafted copolymers. In the case of PHB-*g*-PAA, remelting and forming a thin film allowed enzymes to degrade the polymer, while in the case of PHB-*g*-PVAc, a saponification reaction was needed to transform PVAc into polyvinyl alcohol (PVA) [201,202]. Another disadvantage of graft copolymerization is that the amorphous phase of the polymer undergoes grafting much more easily than the crystalline phase [198,199]. Mitomo et al. investigated the degree of crystallinity of PHB films before and after grafting with MMA, showing that the degree of crystallinity was not affected, regardless of the grafting degree [198]. This suggests that the crystalline part of PHB hardly undergoes copolymerisation with MMA [198,201,203]. Grafting of the crystalline phase should result in a reduction in the proportion of this phase in the polymer, but this was not observed in the case of grafting with MMA [198]. Graft copolymerization proceeding via radical mechanisms is also accompanied by two undesired reactions: degradation of polyester chains and crosslinking [35,197,198,203,204,205].

UV light is non-ionizing radiation that carries too little energy to penetrate deeply, primarily interacting with the surface of the material, unlike gamma radiation [35,206]. Chen et al. performed surface grafting reactions on PHBV films using a method that involved simultaneous UV irradiation and a mixture of benzophenone and a compound marketed under the name Irgacure 907 as photoinitiators [35]. They used *N*-vinylpyrrolidone (VP) as the monomer containing a double bond, resulting in a surface with significantly reduced hydrophilicity compared to the unmodified film. By using the pre-irradiation method, the surface of PHBV films was also successfully grafted with PHEMA, poly(acrylic acid) (PAA), and polyacrylamide (PAM) [206,207,208,209,210,211]; the chemical structures of VP and acrylamide (AM) are shown in Figure 6. Benzophenone was used as the photoinitiator for grafting these polymers. However, none of the PHBV films modified in this way were used for biological studies.

In addition to films, the surfaces of porous tissue scaffolds made from PHBV using the solvent casting and particulate leaching method were also modified with UV radiation [212,213]. These scaffolds were grafted with AM in the presence of benzophenone, resulting in modified materials with improved hydrophilic properties compared to the original ones. The grafted scaffolds were subjected to cytocompatibility tests, which revealed that the introduction of amide groups onto the PHBV surface promoted the adhesion and proper reorganization of the cytoskeleton in already adhered chondrocytes [213]. Unfortunately, the biological studies did not assess the viability of chondrocytes in response to exposure to PAM-modified scaffolds. However, viability tests were conducted using bone mesenchymal stem cells, which showed that the PHBV scaffold grafted with AM supported the proliferation of these cells to a similar extent as the unmodified scaffold [212].

**Figure 7:**
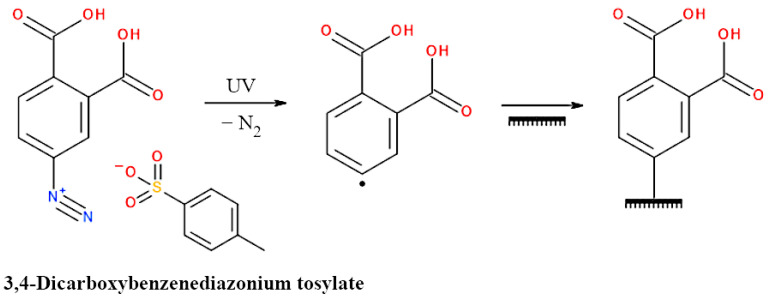
Simplified scheme for chemical surface modification using UV light and 3,4-dicarboxybenzenediazonium tosylate.

Using UV light, it was also possible to modify the surface of electrospun PHB fibers. For this purpose, the fibers were subjected to a reaction with 3,4-dicarboxybenzenediazonium tosylate, which decomposes under UV light, forming an aryl radical [157,214]. Such radicals couple with the main chain of PHB, resulting in a scaffold containing surface carboxyl groups (Figure 7). The presence of these groups made the originally hydrophobic PHB fibers become hydrophilic. Scaffolds modified with the diazonium compound, used in in vitro culture, were found to better support osteoblast adhesion and proliferation compared to unmodified fibers [157].

Surface modification of PHA products through chemical methods to initiate graft copolymerization is relatively uncommon. Nonetheless, benzoyl peroxide was successfully used in the grafting of AM and HEMA onto PHBV surfaces [203,215]. In both cases, it was noted that the grafting process occurred not only on the surface of the film but also in the bulk. In addition, it was shown that grafting the PHBV film with HEMA resulted in improved hydrophilicity [203]. Thermal properties studies by differential scanning calorimetry (DSC) showed that the degree of crystallinity of the modified film decreases with increasing grafting efficiency [203].

Chen and co-workers first grafted methacrylic acid (MAA) (Figure 6) onto electrospun PHBV fibers, using benzoyl peroxide, and then utilized the carboxyl groups introduced by the MAA for covalent binding of quercetin (QUE) to the fiber surface [194]. In this way, they obtained the PHBV-*g*-QUE graft copolymer. The modified fibers were subjected to detailed biological studies, which showed that these chemical modifications had a positive effect on the ability of the fibers to promote chondrocyte proliferation [194].

### 4.2. Degradation Reactions of PHA Chains

Polymer degradation is a reaction in which the bonds in the main chain of macromolecules are broken. In the case of PHA, these reactions are primarily used to obtain their oligomers, which most often act as intermediates in the synthesis of di-, tri- and multiblock copolymers. Degradation reactions do not produce monomers, which is why depolymerization reactions are necessary. The cleavage of ester bonds in the PHA backbone typically occurs through one of two reaction mechanisms: the nucleophilic substitution of the acyl group or β-elimination. Degradation reactions are always accompanied by a decrease in the average molecular weight of the polymer, and often also by a change in the chemical structure of its end units.

The most common reactions for obtaining PHA oligomers from their high-molecular-weight counterparts are nucleophilic acyl substitution reactions. These reactions include hydrolysis, aminolysis, and alcoholysis (transesterification). The structural feature of PHAs that determines their reactivity in the above-mentioned types of reactions is the presence of an electrophilic carbon atom of the ester group in their chemical structure. Nucleophiles such as primary amines and alcohols can add to this carbon atom, giving a tetrahedral intermediate [216]. The intermediate can react further after proton transfer. This reaction results in the release of a leaving group and the formation of two new poly- and/or oligoester chains.

**Figure 8:**
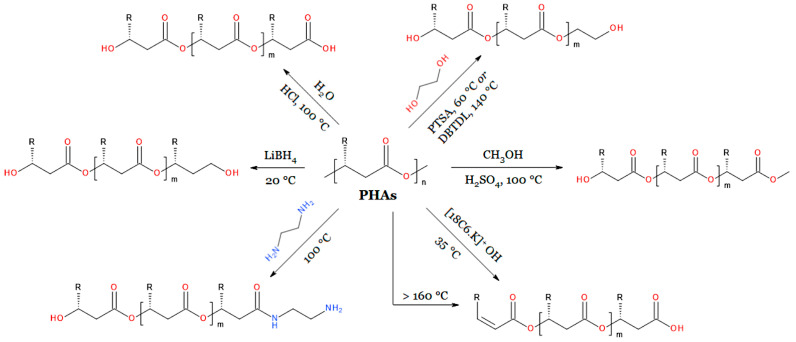
Schematic overview of the degradation reactions to which biopolyesters are subjected in order to obtain their oligomers. For simplicity, only those oligomers that are the main products of the specified reactions are shown; R = hydrogen atom or an alkyl group.

The carbon atom of the PHB ester group is not electrophilic enough to react effectively with water and alcohol molecules even at elevated temperatures [217]. For this reason, hydrolysis and alcoholysis of PHB are carried out in the presence of a catalyst. Carboxylic acids and alcohols are the products of ester hydrolysis. As a result, macromolecules containing a carboxyl and hydroxyl end group should be obtained. In the case of PHA, such macromolecules can be synthesized by hydrolyzing them in an acidic environment (a schematic summary of the degradation reactions used in the synthesis of PHA oligomers is presented in Figure 8). Alkaline hydrolysis is associated with the occurrence of an undesirable β-elimination reaction [218]. Lauzier and co-workers studied the hydrolysis reaction of PHB by carrying it out at the boiling point of 3 M aqueous HCl solution [219]. They showed that this reaction proceeds according to the mechanism of random chain scission. After 14 h of hydrolysis, they obtained oligomers with a number-average molecular weight of 1400 g/mol and a relatively low mass distribution (M_w_/M_n_ = 1.4) [219].

The most common reaction used in the synthesis of PHA oligomers is transesterification. Alcoholysis of biopolyesters is typically performed in two ways. The first method involves dissolving the high-molecular-weight polymer in chloroform and carrying out transesterification at its boiling point (approximately 61 °C) in the presence of p-toluenesulfonic acid (PTSA) as a catalyst [217,220,221,222,223,224,225]. In the second method, diethylene glycol dimethyl ether is used as a solvent, and alcoholysis is conducted at a temperature of 140 °C with an organometallic catalyst, i.e., dibutyltin dilaurate (DBTDL) [151,226,227,228,229]. However, in some cases, the reaction is carried out without the use of any solvent at all [230]. If a low molecular weight monohydroxy compound, such as methanol or 1-hexanol, is used as the nucleophilic agent, transesterification will yield PHA oligomers that theoretically contain only one reactive functional group—a secondary hydroxyl group [228,231]. Monohydroxylated biopolyester oligomers containing an acetate group at one of the chain ends can be obtained by reacting PHA with methanol at 100 °C using H_2_SO_4_ as a catalyst (Figure 8) [154,195,231,232]. Dihydroxyl compounds (diols), on the other hand, lead to oligomers with two different terminal hydroxyl groups (oligoester diols)—a primary and a secondary group (Figure 8) [217]. The most commonly used diols are ethylene glycol and 1,4-butanediol [217,220,224,225,226,229,230,233]. Among triols, glycerol is the most frequently used [217,225]. Transesterification of PHB carried out at the boiling point of chloroform for 6 h with a 10-fold molar excess of ethylene glycol yields oligoester diols with M_n_ = 4000 g/mol and M_w_/M_n_ = 1.5 [217]. Hirt et al. obtained oligomers with M_n_ = 2300 g/mol and M_w_/M_n_ ≈ 2 using diglyme and the same excess of ethylene glycol [226].

To obtain biopolyester oligomers containing hydroxyl end groups, alcoholates are not used as nucleophilic agents. These compounds are sufficiently basic that, in addition to attacking the carbonyl carbon of PHB, they can also abstract a hydrogen atom from the α-carbon, resulting in a β-elimination reaction product—a chain with an unsaturated terminal group (derived from crotonic acid). It can be assumed that the reaction between PHB and a relatively strong base proceeds via the E1cB mechanism [234]. The structural features of PHB support this reaction mechanism: the presence of an acidic α proton, the electron-withdrawing character of the ester group, which stabilizes the carbanion formed after proton detachment, and the separation of the carboxylate ion as a leaving group (weak leaving group) [235,236]. If the leaving group is a weak leaving group, the rate-limiting step in the E1cB reaction is the cleavage of this group from the tetrahedral intermediate [237]. At sufficiently high temperatures, the E1cB elimination reaction is also initiated by weak bases such as sodium or potassium carboxylate [234,238].

PHA degradation in alkaline environments has been the subject of several studies [218,239,240,241,242,243]. As a result, oligomers containing a carboxylate group at one end of the chain and a mer with a double bond at the other end are produced. Iwata and co-workers were among the first to conduct alkaline hydrolysis of PHB, obtaining oligomers with M_n_ = 2600 g/mol and M_w_/M_n_ = 1.5 [239]. The reaction was carried out in a chloroform-5 M aqueous KOH solution system, using 18-crown-6 (18C6) as an auxiliary substance. Crown ethers, by forming stable complexes with metal cations such as sodium or potassium, significantly increase the reactivity (including basicity) of their counteranions [244,245]. The presence of unsaturated and carboxylate end groups in the oligomers obtained from the reaction of biopolyesters with KOH was later confirmed by electrospray ionization mass spectrometry (ESI-MS) [218,241]. These oligomers were used, among other applications, in the synthesis of diblock copolymers with synthetic poly([*R*,*S*]-3-hydroxybutyrate) [240,242].

Oligomers of biopolyesters with unsaturated and carboxyl end groups can be also obtained by the thermal degradation of their high-molecular-weight counterparts [189,246,247,248]. This degradation occurs at temperatures as low as 160 °C and, similar to the alkaline hydrolysis of PHA, is an example of a β-elimination reaction [191]. However, it does not proceed according to the E1cB mechanism but involves the formation of a six-membered ring as a transition state [43,191,247,249]. Thermal degradation is preferably carried out in an extruder (reactive extrusion) using biopolyester and a suitable base (e.g., sodium bicarbonate) [250]. This designed process combines the E1cB reaction with the thermal decomposition of PHA. Using reactive extrusion, Kawalec and co-workers synthesized 3HB oligomers containing an unsaturated (crotonate) and a carboxylate end group [250]. These researchers carried out the degradation in an extruder at a temperature of 170 °C in the presence of a salt of a weak acid and a strong base, i.e., sodium bicarbonate or sodium carbonate, obtaining compounds with M_n_ = 750 g/mol and M_w_/M_n_ = 1.47 after just 15 min.

The presence of a base in the reaction medium significantly accelerates the degradation reaction of bacterial polyesters. Nguyen and co-workers performed the thermal decomposition of PHB and PHBV without using any excipients [246]. They heated the mentioned polyesters to 190 °C, obtaining oligomers with number-average molecular weight in the range of 1200–1600 g/mol after only 9 h of heating. Noteworthy in this method is the high yield of oligomers, which amounts to over 80%. Additionally, by varying the duration of thermal degradation, the average molecular weight of the products can be controlled [248]. Oligomers containing double bonds are very useful in organic synthesis because they can be used in various radical reactions, including polymer grafting processes [189,251].

Amines react with biopolyesters to form oligomers that are products of nucleophilic acyl substitution and E1cB-type elimination reactions [252]. Boyandin and co-workers carried out the aminolysis of PHB by first dissolving it in *N*,*N*-dimethylformamide (DMF) or 1,4-dioxane and then reacting it with ethylenediamine (EDA) or 1,4-diaminobutane; the reaction was performed at 100 °C for 10 h. Among the products of PHB degradation by aliphatic amines, they identified oligomers with a crotonate terminal group (β-elimination reaction products) as well as products of nucleophilic substitution reactions, i.e., oligomers containing a terminal hydroxyl group, an amide group, and a terminal amine group [252]. The reactions were carried out without the use of a catalyst because primary amines are sufficiently nucleophilic to attack the carbon atom of the ester group and abstract the hydrogen atom of PHB in the α-position under appropriate conditions. The oligomer mixture obtained using EDA and DMF was characterized by an average mass of M_n_ = 2100 g/mol and a M_w_/M_n_ ratio of about 1.2.

**Figure 9:**
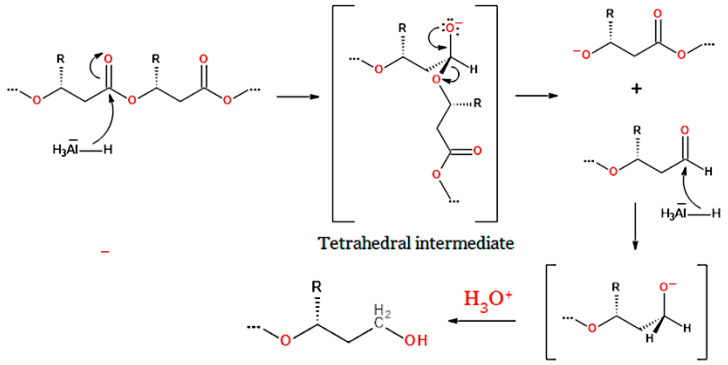
Mechanism of biopolyester reduction using LiAlH_4_ (the counterion has been omitted for clarity); R = hydrogen atom or an alkyl group.

An example of nucleophilic acyl substitution is the reduction of biopolyesters by lithium aluminium hydride (LiAlH_4_) or lithium borohydride (LiBH_4_). This reaction, similar to the transesterification with a diol, leads to the formation of compounds containing two terminal hydroxyl groups. The nucleophile that attacks the carbonyl carbon atom to form a tetrahedral intermediate is the hydride anion (H^−^) in the case of the reaction with LiAlH_4_ (or LiBH_4_) [216]. However, after cleavage of the alkoxide ion from the intermediate formed with H^−^, a new ester group is not obtained, as in the case of alcoholysis of polyesters, but rather an aldehyde group (Figure 9). The newly formed aldehyde group is immediately reduced to a primary hydroxyl group in the next step. Following the reduction reaction, no substitution is observed in the ester group of the PHA ester; rather, there is a transformation of this group into a hydroxyl group.

Kwiecień and co-workers performed the reduction of P34HB using LiBH_4_ as the reducing agent [253]. After dissolving P34HB in tetrahydrofuran (THF), the reduction reaction was carried out at room temperature for 4 h. As a result, they obtained oligomers with M_n_ = 1300 g/mol and M_w_/M_n_ = 1.7, which were terminated with primary and secondary hydroxyl groups. The chemical structure of the synthesized oligomers was confirmed by proton magnetic resonance spectroscopy (^1^H NMR) and ESI-MS mass spectrometry. A limitation of the method developed by Kwiecień is that it can only be effectively applied to biopolyesters that dissolve in specific solvents. Reduction using LiBH_4_ can only be carried out in solvents in which LiBH_4_ dissolves and that are not themselves reduced (such as diethyl ether, THF, or toluene). To avoid competitive and undesirable β-elimination reactions, reduction reactions of bacterial polyesters using LiBH_4_ should not be performed at temperatures higher than room temperature. Both PHB and PHBV have been shown to be insoluble in ether, tetrahydrofuran, and toluene at room temperature. PHB-derived oligomers with relatively low average molecular weight (M_n_ = 2800 g/mol, M_w_/M_n_ = 1.49) that are terminated with functional groups of only one type (hydroxyl groups), can be obtained using LiBH_4_ according to the modified method developed by Chaber et al. [254]. In this method, the reduction reaction is carried out in a heterogeneous system in which PHB powder constitutes the solid phase and the ethereal LiBH_4_ solution constitutes the liquid phase.

Sodium borohydride (NaBH_4_) was also used to degrade bacterial polyesters. Bergamaschi et al. carried out the degradation reaction with NaBH_4_ in the presence of methanol, having previously dissolved PHB in chloroform [255]. The method they developed proved to be effective but not very selective. In addition to oligomers terminated with hydroxyl groups, they also obtained oligomers terminated with a hydroxyl group on one side and a carboxyl group on the other. These oligomers were also produced in relatively large quantities.

Degradation reactions are also used to chemically modify the surface of PHA films [158,169,256,257,258,259,260,261]. These reactions lead to the cleavage of ester bonds and the formation of surface polar groups, which turns hydrophobic biopolyesters into hydrophilic ones [169,258,259,260]. Studies indicate that moderately hydrophilic surfaces promote adhesion and proliferation of various cell types to a greater extent than hydrophobic surfaces [122,262]. In the literature, there are surface modifications of PHB, PHBV, and PHBHHx films that are based on the alkaline hydrolysis or aminolysis reactions.

Karahaliloğlu et al. treated PHB films with an aqueous NaOH solution, generating surface carboxyl groups [258]. These modified film surfaces were shown to better support the adhesion and proliferation of human fibroblasts and osteoblasts than the unmodified PHB surface [258]. García-García et al. introduced amino groups onto the surface of a PHBHHx film by treating it with an aqueous solution of EDA at 50 °C [169]. These groups were then used to covalently link a protein containing the amino acid sequence Tyr-Ile-Gly-Ser-Arg (YIGSR). Using scanning electron microscopy (SEM), they showed that films modified with EDA and the YIGSR sequence induce a more accurate reorganization of the cytoskeleton of porcine urothelial cells following adhesion, compared to cells adsorbed on the surface of unmodified PHBHHx [169].

The heterogeneous reduction method developed by Chaber et al. was used not only to synthesize oligoester diols but also to modify the chemical structure of the surface of products made from PHBV [115]. The advantage of the LiBH_4_ method is that it increases the concentration of surface polar groups under very mild conditions—in a short time (5–20 min) and at room temperature. As a result, the hydrophilicity of mats spun from PHBV solutions was increased without affecting the morphological properties of the fibers that make them up. Viability tests conducted using the MTS method showed that fibers modified with a LiBH_4_ solution at a concentration of 2.5 or 5 mmol/dm^3^ stimulated the proliferation of osteoblast-like SaOS-2 cells three times more strongly than unmodified fibers, after just 5 min of exposure. It is worth noting that the LiBH_4_ method is the first chemical method described in the literature that is not based on free-radical mechanisms and allows for the modification of the surface chemical structure of PHA fibers.

### 4.3. Block Copolymers Containing Blocks Derived from PHAs

Oligomers obtained through the degradation of high-molecular-weight PHAs are most commonly used as macromonomers in various copolymerization reactions. This approach leads to the production of di-, tri-, and multiblock copolymers containing structural segments derived from biopolyesters. The first two of these copolymers are often synthesized to be amphiphilic, consisting of at least one hydrophilic block and one hydrophobic block. To obtain such copolymers, PHA oligomers are usually reacted with PEG [33,263,264,265,266,267]. It is important to emphasize that obtaining a copolymer with PHA-PEG blocks is possible not only through the reaction of oligomers with appropriate functional groups but also by transesterification of a long-chain biopolyester with oligomeric ethylene glycol [268,269]. A characteristic feature of di- and triblock amphiphilic copolymers is that they self-organize in water, forming micelles, among other structures [155,264,266,268,270]. As such, PHA-PEG copolymers are most commonly used as matrices for controlled release systems of active substances [24,155,268]. They are rarely used for the fabrication of tissue scaffolds.

In the synthesis of PHA di- and triblock copolymers, oligomers of other biodegradable polyesters, such as PLA or PCL, are also used [33,154,232,267,271,272]. Wu and co-workers synthesized a triblock copolymer of the ABC type, consisting of blocks derived from PHB, PDLLA, and PCL, respectively (PHB-*b*-PDLLA-*b*-PCL) [232]. They observed that this copolymer undergoes microphase separation: the blocks derived from PHB and PDLLA formed the so-called hard segment, while the block derived from PCL formed the soft segment. DSC and XRD studies showed that the crystallinity of PHB decreases as a result of its incorporation into the triblock copolymer [232]. Furthermore, it was found that the film made from PHB-*b*-PDLLA-*b*-PCL promotes osteoblast flattening and proliferation better than films made from PHB, PDLLA, or PCL alone [232].

**Figure 10:**
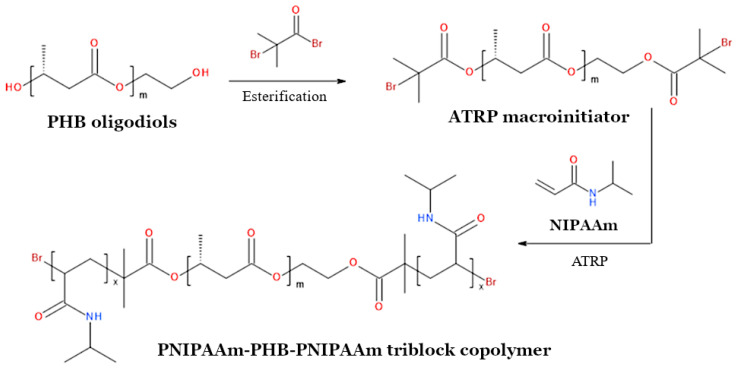
Schematic of the synthesis of thermoresponsive PNIPAAm-PHB-PNIPAAm triblock copolymer via ATRP.

Another example of a triblock polymer used in tissue engineering is a copolymer consisting of a central PHB block flanked by two poly(*N*-isopropylacrylamide) (PNIPAAm) blocks (PNIPAAm-PHB-PNIPAAm). It was synthesized in two steps, using PHB oligodiols as the starting substrates [273]. The first step involved the esterification of the PHB oligomers with 2-bromoisobutyryl bromide, while the second step employed atom transfer radical polymerization (ATRP) of the NIPAAm monomer with the esterified oligomers (Figure 10). The resulting triblock copolymers exhibited thermoresponsive behavior and were used as coating agents for thermally induced cell detachment in tissue engineering. Surfaces coated with PNIPAAm-PHB-PNIPAAm copolymers were effectively used to obtain sheets of embryonic stem cells and human mesenchymal stem cells [274,275].

**Figure 11:**
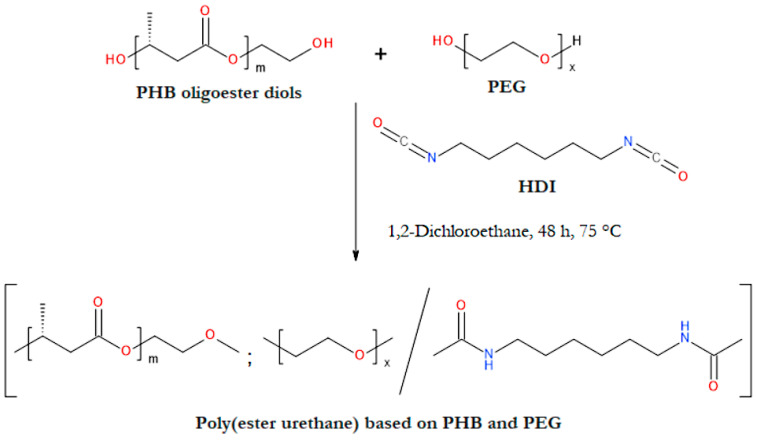
Schematic of the synthesis of poly(ester urethane) from PHB oligoester diols, oligomeric PEG, and HDI [287].

Multiblock copolymers of biopolyesters are most often obtained from their oligomers containing two terminal hydroxyl groups. These oligomers are generally subjected to a one-step reaction with another diol and diisocyanate, resulting in poly(ester urethane)s composed of randomly occurring structural segments derived from PHA and the two other comonomers (Figure 11) [151,222,223,224,230,233,276,277,278,279,280,281,282,283,284,285,286,287]. PHA-based poly(ester urethane)s typically have relatively high M_n_ values, ranging from 25,000 g/mol to over 50,000 g/mol. A distinguishing feature of polyurethanes, compared to other types of polymers, is the microphase separation caused by the incompatibility between the building blocks [151,288]. Blocks derived from scl-PHAs (e.g., PHB and PHBV) usually form crystalline and hydrophobic hard (rigid) segments, while those derived from mcl-PHAs (e.g., PHO) form amorphous and hydrophobic soft (flexible) segments [151,276,285].

**Figure 12:**
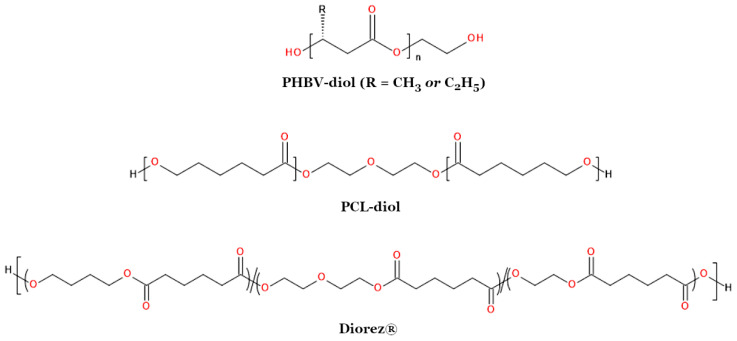
Chemical structure of oligoester diols used in the synthesis of DegraPol polyurethanes.

In the vast majority of cases, when synthesizing poly(ester urethane)s based on PHA, non-toxic hexamethylene diisocyanate (HDI) is used as a coupling agent [151,222,223,224,233,277,278,279,280,281,282,283,284,286,287]. Hirt and co-workers synthesized PHBV-based polyurethanes using two other isocyanates, namely trimethylhexamethylene diisocyanate (TMDI) and L-lysine methyl ester diisocyanate (LDI). However, they demonstrated that the mechanical properties of poly(ester urethane)s are largely independent of the type of chain extender used in their synthesis [276]. In the synthesis, in addition to PHBV oligoester diols (PHBV-diol; M_n_ = 1000 g/mol), they also used other oligomers with hydroxyl end groups to serve as components of the soft segments in the structure of the obtained polymers. These included oligoester diols derived from PCL (PCL-diol; M_n_ = 1200 or 2000 g/mol) and oligoester diols composed of mers derived from ethylene glycol, diethylene glycol, 1,4-butanediol, and adipic acid (Diorez^®^; M_n_ = 1000 g/mol) (Figure 12). Using PHBV-diol, PCL-diol, or Diorez^®^ along with TMDI or LDI in a single-step reaction, they obtained a series of poly(ester urethane)s, which they co-named DegraPol. DegraPol polyurethanes were used for the production of tissue scaffolds, specifically three-dimensional porous foams, which were found to promote the proliferation of chondrocytes and osteoblasts, among others [289,290].

When synthesizing poly(ester urethanes) based on PHB, the following are used as soft segments: PCL oligoester diols, poly(butylene adipate) oligoester diols, poly(diethylene glycol adipate) oligodiols, PEG, poly(propylene glycol), oligoester diols derived from biopolyesters such as PHO, and a hydroxyl-terminated triblock copolymer PCL-*b*-PEG-*b*-PCL [151,223,277,279,280,281,283,284,285,286,287]. Poly(ester urethane)s incorporating blocks derived from PEG are particularly favored by researchers in the scientific community. The introduction of hydrophilic soft segments into the PHB main chain increases its degradation rate and hydrophilicity, with both susceptibility to hydrolytic degradation and hydrophilicity being greater the higher the PEG content in the multiblock copolymer [283,286]. Liu and co-workers produced electrospun fibers from polyurethanes obtained from PHB and PEG and then mineralized them by immersing them in an artificial body fluid [283]. They showed that mineral substances cover fibers made of poly(ester urethane) more than those made from PHBV. Fibers covered with inorganic calcium compounds are used in bone tissue engineering [291].

By changing the molar fraction of individual blocks in the copolymer or their length (average molecular weight), one can determine the effect of the chemical structure of polyurethane on its mechanical and thermal properties [151,226,277,281]. In the case of poly(ester urethane)s based on PHB, it can be concluded that the higher the PHB content in the copolymer, the higher the glass transition temperature of the poly(ester urethane). An increase in the melting point and the degree of crystallinity of the blocks derived from this homopolymer is also observed [151,277,281,285,287]. If the mass fraction of PHB in poly(ester urethane) containing PEG-derived blocks is relatively small (less than 13%), the thermogram obtained by the DSC method does not show the melting of its crystalline phase [151]. It is also worth mentioning that the degree of crystallinity and the melting point of PHB decrease as a result of the poly(ester urethane) formation reaction [151,283].

Certain relationships related to the change in the chemical structure of poly(ester urethanes) based on PHB are also observed in their mechanical properties. Generally speaking, an increase in PHB content in poly(ester urethane) is accompanied by an increase in Young’s modulus and tensile strength, along with a decrease in the elongation at break of the produced poly(ester urethane) film [276,283]. Liu and co-workers, previously mentioned, synthesized a poly(ester urethane) with PHB macrodiols at M_n_ = 1200 g/mol and PEG at M_n_ = 2000 g/mol, resulting in a poly(ester urethane) with M_n_ = 22,700 g/mol, in which the PHB content was about 50% [283]. The film made of such polyurethane had an elongation at break of 1090%, which is more than 100 times higher than that of the PHB homopolymer [283].

Poly(ester urethane)s derived from PHB and PEG, as well as from P34HB and PEG, were found to be hemocompatible and non-toxic to rat aortic smooth muscle cells (RaSMCs), supporting their growth and proliferation [281,282]. They could, therefore, serve as materials for the production of vascular grafts.

In addition to diisocyanates, carboxylic acids and their derivatives are also used as coupling agents. Using these compounds, copolyesters or terpolyesters can be obtained, in which individual mers are connected by ester bonds [152,227,292,293,294]. This is important because ester bonds are highly susceptible to abiotic and enzymatic hydrolysis [295]. Polyesters based on PHA, obtained through polycondensation, are characterized by low average molecular weight; the M_n_ values typically do not exceed 20,000 g/mol and are often even lower than 10,000 g/mol [152,227,292].

Andrade and co-workers synthesized a terpolyester with a molecular weight at peak maximum (M_p_) of 7200 g/mol by using PHB oligoester diols with M_p_ = 3200, PHO oligoester diols with M_p_ = 2100, and terephthalic acid dichloride [227]. Using NMR spectroscopy, they demonstrated that the obtained terpolyester consists on average of two blocks derived from PHO, one from PHB, and two from terephthalic acid. This polymer exhibited good thermoplastic properties, with two melting temperatures (129 °C and 140 °C) and a glass transition temperature of −41 °C [227].

Li and co-workers, using PHB oligoester diols and carboxyl-terminated PEGs as comonomers, prepared a series of PHB-based alternating copolyesters that were characterized by M_n_ values in the range of 7800–18,900 g/mol [152]. The copolymerization was carried out at room temperature for two days, by dissolving the substrates in advance in methylene chloride. As auxiliary reagents, they used 4-dimethylaminopyridine (nucleophilic catalyst) and *N*,*N*′-dicyclohexylcarbodiimide (condensing agent). Copolyesters of PHB with PEG exhibited a lower degree of crystallinity and a lower melting point compared to the original PHB oligoester diols [152]. By varying the average molar mass of the starting oligomers, these researchers showed that the lower the PEG content of the copolymer, the higher the melting point and crystallinity of the PHB-derived block. From the obtained copolyesters, they produced thin films, which they subjected to the study of the wetting angle. It turned out that the increase in PEG content in the copolymer is accompanied by an increase in the hydrophilicity of the film surface [152].

An increase in melting point, degree of crystallinity, and glass transition temperature as a result of increased PHA content in polyester was also observed by Kwiecień and co-workers [292]. These researchers synthesized terpolyesters from PHBHHx oligoester diols and sebacyl dichloride through melt polycondensation at 120 °C. From one of these polyesters, they produced a porous tissue scaffold using the solvent casting and particulate leaching technique. This scaffold was found to be non-toxic to the HEK 293 cell line and human fibroblasts [292].

## 5. Challenges and Future Perspectives

Nowadays it is important to design and implement technological processes in a sustainable and economical fashion. The growing problem of environmental pollution and extensive exploitation of non-renewable resources highlight the urgent need for green chemistry solutions. However, none of the chemical methods presented in this work were performed in a sustainable or cost-effective manner. Many of the described methods were conducted under harsh conditions and involved the use of halogenated solvents and hazardous compounds, such as isocyanates and acid chlorides. In the near future, methods for the chemical structure modification of PHAs are expected to utilize less toxic chemicals and renewable substrates or to employ procedures that enable the recovery and reuse of certain starting materials.

For a chemical method to be considered sustainable, it is also crucial to consider the costs of PHA production. And the costs of PHA production are relatively high—three times greater than those of petrochemical plastics [29]. The factors affecting the costs of PHA production are related to their biosynthesis in bacteria and will not be discussed in detail here. There is extensive literature on this topic [296]. Reducing these costs could make the scenario in which PHAs replace petroleum-based plastics a reality. This would significantly address the problems associated with environmental degradation. This would also be highly relevant to the chemical modification methods of PHAs, as microbial polyesters would become more widely utilized. The demand for more efficient methods of modifying the chemical structure of biopolyesters would be much greater, thereby accelerating the development of this field.

Regarding the surface modifications of PHA materials, there is a lack of methods that allow chemical alterations of electrospun fibers. The fibrous materials are one of the most promising candidates for scaffold fabrication, as they closely resemble the structure of the native extracellular matrix [297]. Existing surface modification methods of electrospun PHA scaffolds are almost entirely based on radical reactions and, as such, present some disadvantages. One of the issues is that polymers modified by radical reactions often exhibit a lower resorption rate, which is especially detrimental to microbial polyesters. Another issue is that grafting reactions proceed much faster in amorphous than in crystalline polymer regions. This may result in scaffolds with a non-uniform surface. The only non-radical chemical method for fiber modification is based on borohydride reduction, which is a reaction that also occurs in the bulk phase of the material (based on unpublished data). This limits the applicability of this method. Therefore, new mild methods based on non-radical reactions are anticipated to be developed in the future.

Synthesis and exploitation of the PHA-based multiblock copolymers also present some challenges. Typically, PHA-based copolyesters and polyurethanes are synthesized for tissue engineering applications. The problem with the former is that they exhibit low molecular weights (M_n_ less than 20,000 g/mol) and low viscosity values. As such, they are hardly processable through traditional methods such as solvent casting, electrospinning, and injection molding. This limits the applicability of the synthetic PHA-based copolyesters in tissue engineering. On the other hand, synthetic PHA-based polyurethanes contain urethane bonds, which are highly resistant to enzymatic and hydrolytic degradation [298]. As mentioned earlier, one of the disadvantages of microbial polyesters is their slow in vivo degradation rate. Therefore, the synthesis of PHA-based poly(ester urethane)s does not solve this problem, and may even worsen it. Consequently, one can expect that the PHA-based copolyesters of relatively high molecular weights will be synthesized and exploited as scaffold materials in the future.

Several examples describing the synthesis of PHA-based copolymers are presented in the main part of this review. However, many of the described copolymers have not been subjected to biological studies to evaluate their suitability for applications in tissue engineering. More biological tests are needed to assess the full potential of synthetic PHA-based copolymers as scaffold materials. This issue should be addressed in the near future.

## 6. Conclusions

PHAs, a group of naturally occurring polyesters, are gaining increasing attention for their potential as tissue engineering scaffolds. However, microbial polyesters must fulfill numerous requirements to be successfully utilized as scaffold materials. Fortunately, PHAs fulfill the most critical requirements due to their biocompatibility and degradability in physiological conditions. The remarkable biocompatibility of microbial polyesters results partly from the fact that oligomeric PHB (known in the literature as cPHB) and the end product of its in vivo degradation, namely, 3-hydroxybutyric acid (3HB), are commonly found in many animal tissues. cPHB forms ion channels in the cell membrane of eukaryotic cells, while 3HB is classified as a ketone body. Unlike lactic acid, a degradation product of PLA, 3HB is not acidic enough to cause chronic inflammation and necrosis. The in vivo implantation of PHA materials is accompanied by a foreign body reaction, but this immune response often subsides quickly, leading to the acceptance of the implant. Consequently, PHA implants elicit a weaker immune response than those made from PLA, PLGA, and PCL, that is, from the polyesters more typically used in tissue engineering than biopolyesters.

Despite their favorable properties, microbial polyesters are not widely used as scaffold materials due to several inherent limitations. Many studies have shown that mammalian cells prefer moderately hydrophilic surfaces for their attachment, proliferation, and growth. However, PHAs are not hydrophilic but hydrophobic. Moreover, because PHB and PHBV, the most prominent members of the PHA family, are stiff and inflexible, their scaffolds can cause chronic inflammation upon implantation. This is especially true if these scaffolds are used for soft tissue regeneration. PHAs also suffer from poor resorption rates, which are often too slow to match the formation rate of the tissue being the therapeutic target. Chemical methods used for tissue engineering purposes aim to overcome these limitations.

Generally, the chemical methods used for structure modification of PHAs may be divided into two groups: those that modify the surface chemistry and those that alter the bulk chemistry of biomaterials. In particular, surface modification methods are commonly used during the scaffold preparation process. This is because these methods are relatively simple and easy to employ, but at the same time, they are very effective in improving cell-scaffold interactions. Probably the most essential surface property of scaffolds is their hydrophilicity. Since PHAs are hydrophobic, most chemical methods aim to make the surface of their scaffolds more hydrophilic.

Four types of chemical reaction have been used to modify the surface chemistry of PHA scaffolds: aminolysis, alkaline hydrolysis, borohydride reduction, and graft copolymerization. Usually, the surface of scaffolds made from PHA polymers is enriched with hydroxyl groups. These groups can be introduced into the surface of PHA scaffolds through base hydrolysis with KOH, a reduction reaction with LiBH_4_, and a graft copolymerization reaction using HEMA and HEAA. Generally, the PHA surfaces enriched with polar groups show increased wettability and improved cell-material interactions. Most of the chemical methods have been employed to alter the surface properties of PHA films. Only borohydride reduction and graft copolymerization are used to improve the biological performance of PHA electrospun fibers. These two types of reaction can proceed under very mild conditions, which is crucial for maintaining the morphological features of the fibers. The surface-modified PHA scaffolds have been used to enhance the viability and proliferation of such cells as fibroblasts, osteoblasts, chondrocytes, tenocytes, adipose-derived stem cells, and bone mesenchymal stem cells.

It is impossible to enhance the mechanical properties of microbial polyesters by modifying only their surface chemistry. Modifications in bulk chemistry of PHAs are the subject of the second group of methods. These methods are based on obtaining PHA oligomers and using them to synthesize their multiblock copolymers. By employing such approaches, various monomers can be incorporated into the PHA chain. The most commonly used comonomer in the polymerization of PHA oligomers is PEG. It is usually used to prepare polyesters and polyurethanes containing PHB blocks. In general, copolymers of PEG and PHB exhibit lower crystallinity, improved mechanical properties, a faster degradation rate, and increased hydrophilicity than the homopolymer of 3HB. Therefore, they are more suitable as scaffold materials for tissue regeneration. Such synthetic copolymers of PHB have been successfully used to promote the survival and proliferation of osteoblasts, chondrocytes, and fibroblasts. The other way to modify the bulk composition of PHAs is to carry out grafting reactions to prepare their graft copolymers. For example, PHB and cellulose components of the graft copolymer both showed lower crystallinity than their separated counterparts. It should be noted, however, that graft copolymerization is much more frequently used to modify the surface properties of PHA scaffolds than to alter their bulk chemical compositions. Improvements in the mechanical properties of PHAs are usually achieved through the methods described at the beginning of this paragraph, that is, through the polymerization of the PHA oligomers with carefully selected monomers.

Certainly, PHAs have many advantages that make them very promising for use in tissue engineering applications. However, they are not free of drawbacks and limitations. Fortunately, researchers have developed many chemical methods that allow these limitations to be overcome. As a result, PHAs are still finding applications in different areas of regenerative medicine.
